# Fluorescence Microscopy Visualization of Contacts Between Objects**

**DOI:** 10.1002/anie.201410240

**Published:** 2015-01-28

**Authors:** Tomislav Suhina, Bart Weber, Chantal E Carpentier, Kinga Lorincz, Peter Schall, Daniel Bonn, Albert M Brouwer

**Affiliations:** van't Hoff Institute for Molecular Sciences, University of AmsterdamP.O. Box 94157, 1090 GD Amsterdam (The Netherlands); Institute of Physics, University of AmsterdamP.O. Box 94485, 1090 GL Amsterdam (The Netherlands)

**Keywords:** fluorescent probes, interfaces, mechanical properties, surface chemistry

## Abstract

The area of contact between two objects was detected by using the strong enhancement of the fluorescence of rigidochromic probe molecules attached to one of the surfaces. Confinement of the molecules suppresses nonradiative decay and turns on the fluorescence. The approach is demonstrated by imaging of the contact area of a plastic sphere in contact with a flat glass surface. Our results agree excellently with the prediction of Hertz’s classical theory based on elastic deformation.

The study of contact mechanics dates back to 1882 with the publication of “*On the contact of elastic solids*” by Hertz.[1] For the behavior of virtually all mechanical systems, the mechanics of the contact between their constituents is crucial. Friction, for instance, is a direct consequence of contact mechanics and is responsible for about 30 % of the world energy consumption.[2] Surprisingly little is known about how the physical contacts between objects arise, although this is essential for understanding their mechanics.[3] The main challenge is that since most (if not all) surfaces possess a certain roughness, the actual contacts may occur on microscopic length scales, even for large macroscopic bodies. Bowden and Tabor were the first to emphasize the importance of surface roughness for bodies in contact.[4]

Herein we describe the first direct visualization of mechanical contacts at the microscale by means of fluorescence microscopy, using specifically developed probe molecules that fluoresce when confined in a contact. To achieve this goal we synthesized rigidochromic fluorescent molecules that fluoresce only very weakly in (low-viscosity) solutions owing to the presence of rapid non-radiative relaxation pathways for the excited state.[5–7] This fast non-radiative decay is triggered by the rotation around a specific bond in the molecule. When the rotation of the bond is hindered, the non-radiative decay is suppressed, and the excited state decays by emitting a photon. When rigidochromic molecules are incorporated in a very viscous medium, such as a glassy polymer matrix, a strong fluorescence is observed. This effect has been used to measure local viscosities in polymer films and study their free volume and glass transition,[2,^5^,^7^,8] and to investigate the viscosity of membranes and intracellular media.[3,^9^–11] We show that the confinement between two surfaces also impedes the non-radiative relaxation of the probe molecule **1** that starts fluorescing strongly when confined. This effect then allows the detection of the physical contacts between surfaces on a molecular scale.

For our experiments, we synthesized a new member of the DCDHF class of compounds that has in recent years been developed by Moerner, Twieg, and co-workers for single-molecule imaging.[8,^9^,^11^,14] (**1**, Scheme 1; for details, see the Supporting Information). This chromophore has advantages over previously used viscosity sensitive probes such as dicyanovinyljulolidines[6,[7,[15] and BODIPY dyes:[10,^16^,17] excitation and emission in the visible part of the spectrum, good photostability,[8] and particularly low fluorescence in low-viscosity polar solvents. Compound **1** was chemically linked to the surfaces of glass coverslips to investigate the imaging of contact areas. Compound **2** was used for comparison.

**Scheme 1:**
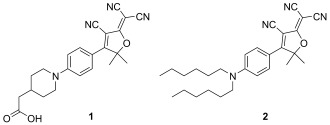
The compounds investigated in this study.

As a first step to characterize their photophysical properties, we measured absorption and emission spectra of compounds **1** and **2** in a series of solvents. The data listed in the Supporting Information (Table S1) show that there is little difference in the properties of **1** and **2**, as expected. Both show a weak solvatochromic effect in absorption and in emission. The fluorescence quantum yields *Φ*_f_ are low, and tend to decrease with increasing solvent polarity. Fluorescence decay times *τ*_f_ follow the same trends as the quantum yields. In some solvents they were shorter than the time resolution of our instrument (<10 ps). The quantum yields and decay times are larger in solvents of higher viscosity. For example, in cyclohexanol *τ*_f_=0.46 ns, and *Φ*_f_=0.11, while in 2-propanol *τ*_f_=0.040 ns and *Φ*_f_=0.010. The reason for the difference is that in low-viscosity solvents rapid nonradiative deactivation of the excited states occurs by twisting of the exocyclic C—C(CN)_2_ bond, as was reported previously for DCDHF chromophores.[9] In some solvents we found a bi-exponential fluorescence decay, indicating that the photophysical behavior of this chromophore is more complicated than was suggested previously.[9]

We systematically studied the effect of solvent viscosity with minimal effect of polarity by subjecting solutions of compound **1** in acetonitrile to different hydrostatic pressures. To convert the hydrostatic pressures to changes in viscosity, we used the relationship between viscosity of acetonitrile and pressure described by Martin et al.[18] using data from Dymond et al.[19] The results are shown in Figure 1.

**Figure 1:**
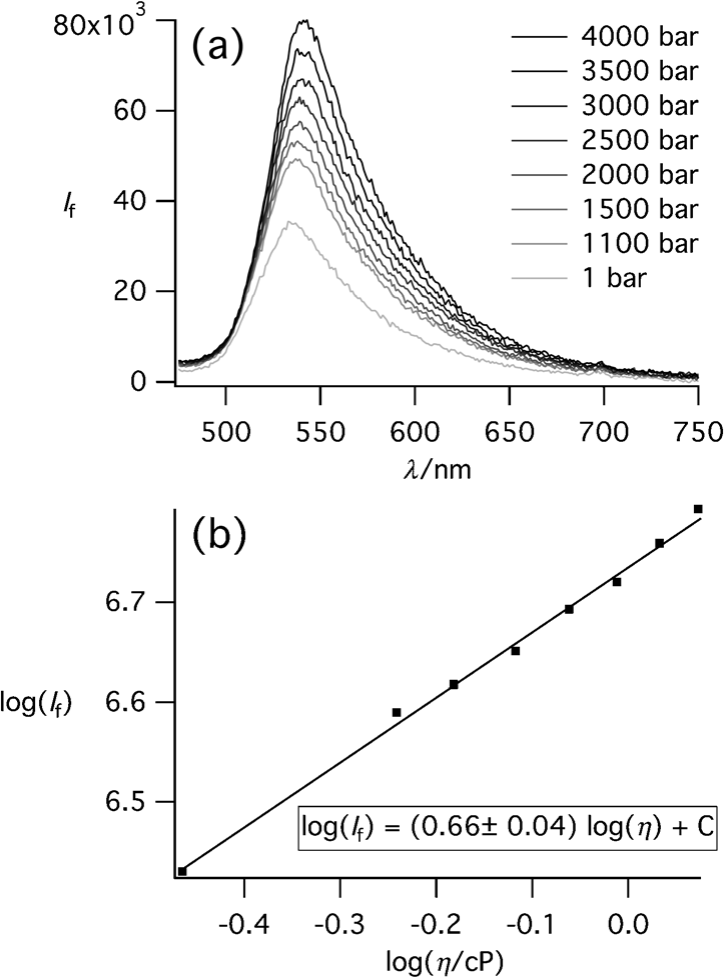
a) Fluorescence spectra of 1 in acetonitrile at different pressures. b) Fit of the intensity data according to Equation (1).

We find that the fluorescence intensity can be described well by the Förster–Hoffmann equation:[12]

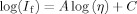
(1)

In Equation (1), *A* is a constant that depends on the dye and the solvent.[15] From the slope of the line shown in Figure 1 b we find *A*=0.66±0.04. For other systems with the same type of rotor unit, values of *A* between 0.5 and 1.2 were recently reported, depending on the nature of the solvent.[4,[15]

To be able to look at the contact of an object with a flat surface, we covalently attached probe **1** to glass cover slips. The latter were functionalized with *N*-(2-aminoethyl)-3-aminopropyl-trimethoxysilane (Supporting Information, Scheme S1) and the dye was attached using an amide bond. Fluorescence emission and excitation spectra of surface-bound **1** (Supporting Information, Figure S2) were found to be very similar to those of **1** and **2** in solution. The absence of broadening of the bands shows that aggregation of surface-bound dye molecules does not occur or has no significant effect on the electronic structure of the chromophore.

On the other hand, the fluorescence lifetime of the bound molecules is quite different. The fluorescence decay was measured at several locations on air-dried cover slips using the single photon timing unit of a confocal microscope. The time profiles were fitted using a double exponential function (Supporting Information, Equation S1). A slow decay component (*τ*_1_=1.4±0.2 ns) was found to be present in addition to a faster one (*τ*_2_=0.36±0.04 ns). The average lifetime, for each point (Supporting Information, Equation S2), amounted to *τ*_av_=0.7±0.2 ns. The deviation from single exponential decay can be attributed to spatial heterogeneity: the surface-bound probe may exist in different local environments, in which the molecules have different nonradiative decay rates.

The quantitative measurement of fluorescence intensities of dye monolayers is difficult owing to the very weak absorption. Therefore we use the average lifetime to quantify the fluorescence intensity of the dye on the cover slip.[20,[21] The quantum yield is expected to be linearly dependent on the lifetime as *Φ*_f_=*τ*_av_ *k*_f_, where *k*_f_ is the radiative decay rate constant of the chromophore. The values of *Φ*_f_ and *τ*_av_ for compound **1** in several solvents (Supporting Information, Table S1) give *k*_f_=0.24±0.06 ns^−1^. We do not observe a systematic dependence of *k*_f_ on solvent polarity, and we assume that it does not change significantly when the dye is bound to the surface. Then, based on *τ*_av_=0.7 ns for cover slips functionalized with rigidochromic probe **1**, we can estimate the fluorescence quantum yield to be about 0.17. Thus, on the cover slip, the emission of the probe is considerably stronger than in solution, but weaker than reported for **2** in the PMMA matrix. This is because the surface-bound probe molecules interact strongly with the surface, reducing the freedom of intramolecular rotation.

To obtain a suitable dynamic range for the rigidochromic effect, we immersed the slides in DMSO. This led to a clearly weaker emission, because the chromophore is solvated and free to undergo rotational motion in the excited state. The lifetime is reduced to *τ*_av_=0.31±0.02 ns, corresponding to a fluorescence quantum yield of about 0.07. Thus, although the fluorescence is still stronger than in the solution, the nonradiative decay is faster than on the air-dried cover slips.

We generated contacts of spherical poly(methyl methacrylate) (PMMA) beads pressed onto the probe-functionalized cover slip, in contact with DMSO. A force transducer was used that exerts and records a well-defined force. Fluorescence was excited and detected through the cover slip, using an epifluorescence confocal microscope. The DMSO serves a dual purpose in these experiments: it not only reduces the fluorescence intensity before the contact is established but also provides a sufficient matching of the refractive indices of the glass and PMMA to avoid the effects of refraction of light at the interfaces.

When the bead is pressed onto the cover slip, the confinement leads to a clear fluorescence increase owing to the rigidochromic effect: a roughly circular fluorescent spot appears and increases in size as the force is increased (Figure 2). When the bead is retracted and placed again with the same load, the contact area is reproduced within ±5 %.

**Figure 2:**
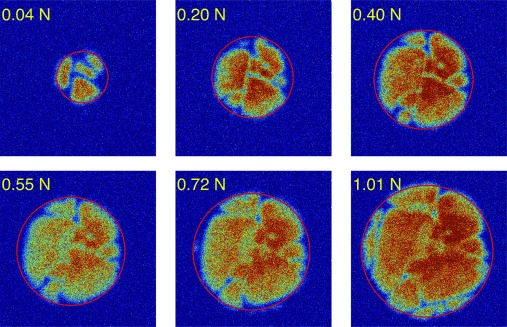
Representative fluorescence intensity images with the focal plane positioned at the surface of a cover slip with covalently linked dye 1. A PMMA bead is pressed on the cover slip with the indicated loads, resulting in an increase in the contact area in which the fluorescent probe lights up. The size of the images is 200 μm×200 μm.

To compare with the classical Hertz theory, which was exactly devised for this situation,[1,[22] we estimated the macroscopic contact area by fitting a circle to the fluorescent area. In Hertz theory, the radius *a* of the contact area between a sphere (radius *R*) and a flat surface pressed against each other with force *F* is described by:

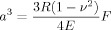
(2)

In Equation (2), *E* is the Young’s (shear elastic) modulus and *ν* is the Poisson ratio of the sphere material (ν=0.37 for PMMA). The modulus of glass can be ignored because it is much higher than that of PMMA. By relating the area to the load according to Equation (2), we can derive the Young’s modulus of the PMMA sphere (Figure 3). The value found *E*(PMMA)=2.0 GPa is a bit lower than the literature value for bulk PMMA, which is presumably due to a slight softening of the PMMA sphere by DMSO.[23] Most importantly, we observe that the theory agrees remarkably well with the experiments, which strongly supports the validity of using immobilized compound **1** as a probe for mechanical contact.

**Figure 3:**
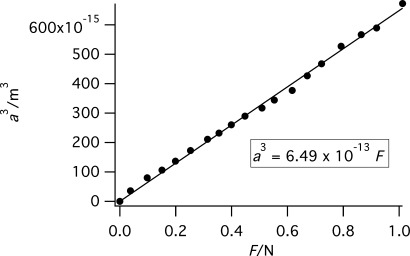
Radius of contact area observed in the fluorescence images (examples in Figure 2) as function of the normal force according to Equation (2).

We note that the fluorescent spot is not perfectly circular, and shows a significant amount of structure within it, implying that there are many small contacts at the microscopic scale, rather than one large homogeneous contact, as is commonly assumed in contact mechanics. At the same time, contact mechanics has been tested many times, and seems to be valid even when the microscopic structure of the contact is not taken into account. This presumably holds as long as the typical scale of the roughness is much smaller than both the radius of the bead and the contact area,[6,[24,[25] which is the case for this experiment.

In summary, the present approach offers a unique method to directly observe the detailed structure of the contact area between two surfaces. We obtain diffraction-limited resolution in the imaging plane, but the resolution in the axial direction is determined by the thickness of the monolayer of dye molecules on the flat glass surface (roughness <1 nm). We are currently investigating the application of this new method to different problems in contact mechanics.
