# Supplementary material for: Fluorescence Microscopy Visualization of Contacts Between Objects
Source: Angew Chem Int Ed Engl. 2015 Jan 28;54(12):3688–91. doi: 10.1002/anie.201410240 (PMC4471612; doi:10.1002/anie.201410240)
Supplement: Supplementary file 1 [file anie0054-3688-sd1.pdf]

Angewandte Chemie
Eine Zeitschrift der Gesellschaft Deutscher Chemiker

Supporting Information

Fluorescence Microscopy Visualization of Contacts Between Objects**

Tomislav Suhina, Bart Weber, Chantal E. Carpentier, Kinga Lorincz, Peter Schall, Daniel Bonn, and Albert M. Brouwer**

anie_201410240_sm_miscellaneous_information.pdf

1. Experimental details

1.1 Instruments

Nuclear magnetic resonance spectra (^{1}H NMR and ^{13}C NMR) were determined using a Bruker ARX 400 (^{1}H: 400 MHz, ^{13}C: 100 MHz) at a temperature of 25 °C. Mass spectra were recorded using Fast Atom Bombardment (FAB) ionization on a JEOL JMS SX/SX102A four-sector mass spectrometer, coupled to a JEOL MS-MP9021D/UPD system, equipped with a Xenon primary atom beam.

UV/Vis absorption spectra were recorded on a Shimadzu 2700 spectrometer. Steady state fluorescence was measured on a Spex Fluorolog 3-22. Fluorescence was detected in the right angle mode, using a concentration where the absorbance at the excitation wavelength λ_{exc} was low (< 0.1), using 1 cm quartz cuvettes. All solvents were of spectroscopic grade or HPLC grade. Fluorescence quantum yields were measured using the relative method, using coumarine 153 as a reference compound ($\Phi_f = 0.53$).[1] The optical high-pressure setup was described in detail in ref. [2]. Briefly, it consists of a stainless steel block equipped with four sapphire windows. A 5 × 5 mm quartz cell containing the sample solution is placed in the middle of the block at the crossing point of the two optical paths. The cell is closed with a movable cylindrical teflon piston, and liquid pressure (n-heptane) is applied to the system using a high pressure screw piston pump capable of generating pressures up to 4.5 kbar. The cell fits in the sample chamber of the fluorometer, and the spectra are measured in the usual right angle mode.
Fluorescence decay curves of **1** were measured using time-correlated single photon counting. For excitation, we used a Titanium:Sapphire laser (Chameleon Ultra-II, Coherent), operating at a repetition rate of 80 MHz, with pulse widths of ~150 fs. The excitation wavelength of 486 nm was obtained by frequency doubling. The instrument response function (FWHM ~ 20 ps) was determined using scattered light at the excitation wavelength from a ludox sample. A Carl Zeiss M20 monochromator was used to select the detection wavelength, and a 488 nm notch filter was used to eliminate excitation light before the MCP detector (Hamamatsu R3809U-50). TCSPC histograms were recorded using ~10^4 counts in the peak channel, and were fitted using non-linear least squares with IRF deconvolution using the DecFit software by Nikolai V. Tkachenko.

Fluorescence decay curves of the monolayer of **1** on cover slips were measured with a MicroTime 200 confocal microscope (PicoQuant GmbH) with a 100× 1.4 NA objective (UplanSApo, Olympus), mounted on a piezo-scanning stage (Physik Instruments GmbH). A detection pinhole with a diameter of 50 μm was used. The laser used for excitation was the same as that used for TCSPC experiments. Time correlated single photon counting histograms were recorded using on average 4 × 10^6 photons. Total decay curves were biexponentially fitted for each measurement using a Maximum Likelihood Estimation method, and deconvolution with a calculated Gaussian IRF was applied. The width of the IRF was ~ 330 ps.
The images shown in Figure 2 in the main text were obtained using a confocal microscope (Zeiss Axiovert 200M) and a microscope control system (Zeiss LSM 5 LIVE). Excitation was at 488 nm. The objective used was a 63 × 1.3 NA (LD A-Plan, Zeiss). An Anton Paar DSR 301 rheometer was used to apply and control the normal force. The plastic beads were 4 mm Clear Acryclic from The Precision Plastic Ball Company, Addingham, West Yorkshire, UK. Images were subjected to smoothing, and

after setting a threshold, contact pixels were identified. In order to determine the contact radii, circles were fitted to the contact areas.

1.2 Synthesis

Compound **1** was prepared following the procedures described by Twieg, Moerner, and coworkers for related compounds.[3-5] We used 4-piperidine acetic acid as the amine in the last coupling step (Scheme S1). Subsequently, the dye was attached to amino-functionalized glass surfaces.

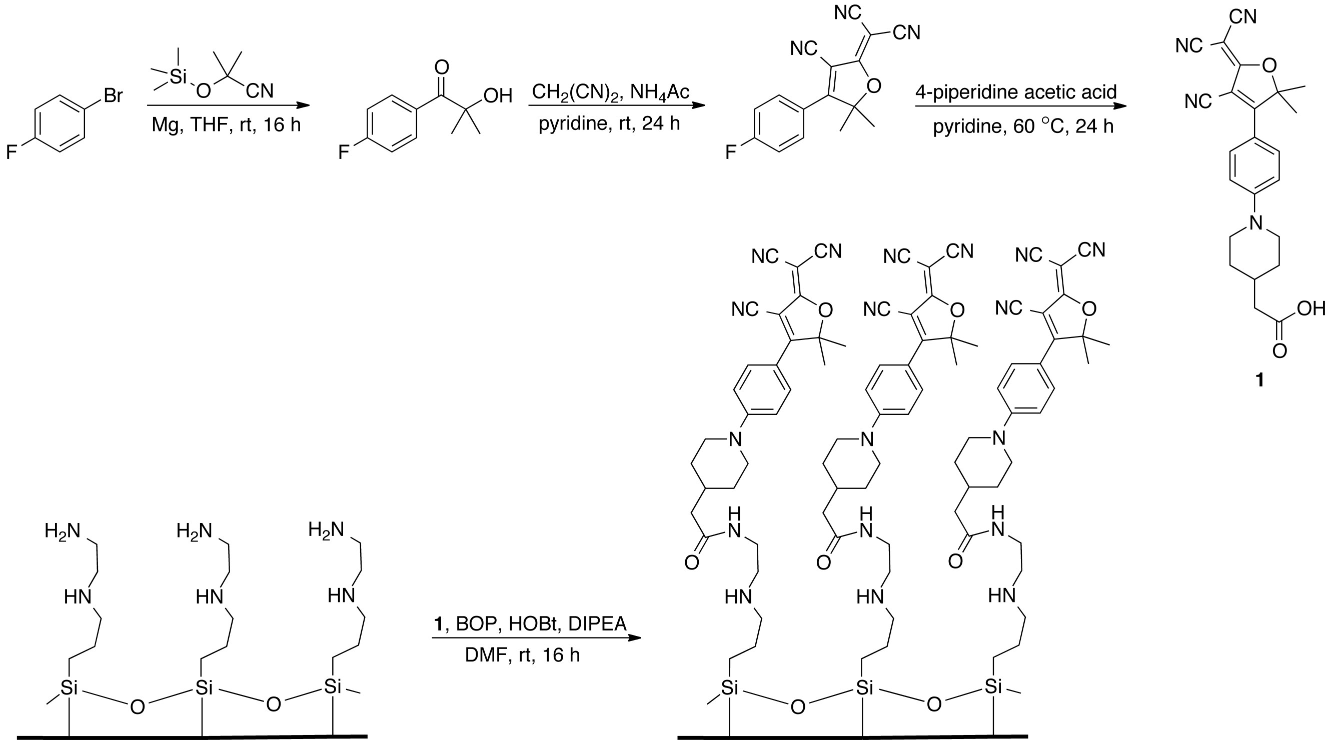

Scheme S1. Synthesis of probe molecule **1** and its attachment to glass surfaces.

*Synthesis of 2-(1-(4-(4-cyano-5-(dicyanomethylene)-2,2-dimethyl-2,5-dihydrofuran-3-yl)phenyl)-piperidin-4-yl)acetic acid (**1**)*

2-(3-Cyano-4-(4-fluorophenyl)-5,5-dimethylfuran-2(5H)-ylidene)malononitrile[3] (0.98 g, 3.5 mmol, 1 eq) and 4-piperidine acetic acid (1.5 g, 10.5 mmol, 3 eq) were dissolved in pyridine (10 mL). The reaction mixture was stirred at room temperature for 24 h. The reaction mixture turned red during this time. It was poured into 200 mL of cold water and left standing in the refrigerator overnight. The precipitate was filtered and purified via flash chromatography (gradient of dichloromethane (DCM) to DCM/MeOH 10:1). Compound **1** was isolated in 26 % yield (0.36 g, 0.89 mmol).

^{1}H NMR (400 MHz, THF-d_8): δ (ppm) = 10.77 (bs, 1H), 8.07 (d, J = 9.4 Hz, 2H), 7.07 (d, J = 9.4 Hz, 2H), 4.15 (d, J = 13 Hz, 2H), 3.07 (m, 2H), 2.21 (d, J = 6.9 Hz, 2H), 2.07 (m, 1H), 1.89 (d, J = 11.6 Hz, 2H), 1.83 (s, 6H), 1.31 (m, 2H).
^{13}C NMR (100 MHz, THF-d_8): δ (ppm) = 177.79, 174.66, 173.39, 155.11, 133.16, 115.34, 114.06, 113.62, 113.28, 112.24, 98.58, 93.39, 47.83, 40.83, 33.88, 32.44, 27.14.
MS (FAB^{+}) MH+: 403.2 (calculated), 403.2 (observed).

Cover Slip Silanization

Cover slips were washed in 3% Hellmanex III solution by sonication for 30 min at 40 ºC and sonicated in deionized water for 10 min and in EtOH for 30 minutes. The cover slips were dried in an oven at 110 ºC for 1 h and further cleaned in an ozone photoreactor for 2 hours. Cover slips were silanized with 2% (volume) *N*-[3-(trimethoxysilyl)propyl]-ethylenediamine (AEAPTMS) in 96% EtOH in which 2% (by volume) of H_2O was added. The pH of this solution was adjusted to ~ 5 by addition of acetic acid. A teflon rack with cover slips was kept for 30 minutes in this solution with stirring. The cover slips were afterwards sonicated three times in EtOH (20 min), washed with acetone and DCM, dried in air and put in an oven for 1 h at 110 ºC. Some cover slips were silanized according to the procedure reported by Basabe-Desmonds et al., [6] but we did not observe any significant difference.

*Immobilization of **1** on glass*

Rigidochromic compound **1** (5 mg, 0.012 mmol, 1 eq), (benzotriazol-1-yloxy)tris(dimethylamino)-phosphonium hexafluorophosphate (BOP) (16 mg, 37 µmol, 3 eq), *N*-hydroxybenzotriazole (HOBt) (5.3 mg, 37 µmol, 3 eq), and diisopropylethylamine (64 µl, 0.37 mmol, 10 eq) were added to the silanized cover slips in DMF (60 mL). The reaction mixture was stirred for 16 h at room temperature. After completion of the reaction, the glass cover slips were removed from the reaction mixture, sonicated in ethanol three times (60-120 min), and rinsed with DCM.

2. Photophysical characterization of DCDHF rigidochromic probes

In figure S1 we show representative spectra of **1** and **2** in different solvents. In Table S1 the photophysical data of the compounds are presented. Fits of the time-resolved fluorescence signals measured using Single Photon Counting were done using a bi-exponential model (eq. S1) when a single-exponential fit gave unsatisfactory results. The average decay time was then calculated using eq. S2. Using this definition of the average lifetime (or average photon arrival time) we can relate it to the quantum yield of the fluorescent layer on the cover slip (eq. S3), with the commonly made assumption that the radiative rate constant k_f is the same for all dye molecules independent of their chemical environment.[7-9]

$$I(t) = A_1\exp(-t/\tau_1) + A_2\exp(-t/\tau_2) \quad \text{(eq. S1)}$$

$$\tau_{av} = (A_1\tau_1^2 + A_2\tau_2^2) / (A_1\tau_1 + A_2\tau_2) \quad \text{(eq. S2)}$$

$$\Phi_f = \tau_{av}k_f \quad \text{(eq. S3)}$$

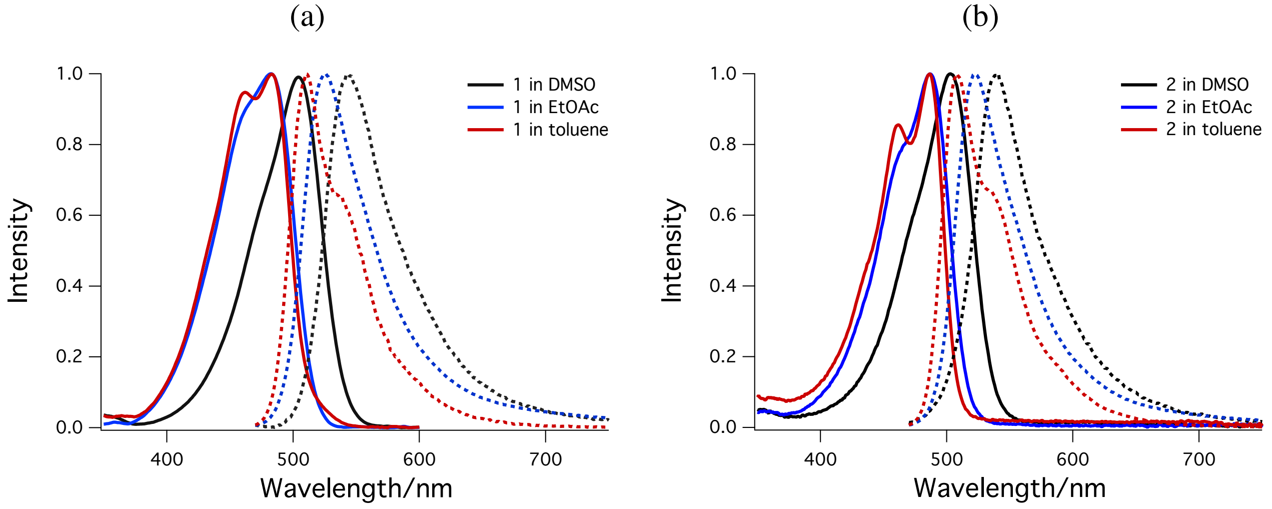

Figure S1. (a) Normalized absorbance (solid line) and emission (dashed line) spectra of **1**; (b) Normalized absorbance (solid line) and emission (dashed line) spectra of **2**; λ_{exc} = 450 nm.

Table S1. Photophysical properties of **1** and **2** in a series of solvents.

Solvent	η^a (mPa·s)	Compound **1**						Compound **2**			
		$\lambda_{abs}{}^b$ (nm)	$\lambda_{em}{}^c$ (nm)	$\Phi_f{}^d$	τ_{f1} $(A_1)^e$ (ns)	τ_{f2} $(A_2)^e$ (ns)	$\tau_{av}{}^f$ (ns)	$\lambda_{abs}{}^b$ (nm)	$\lambda_{em}{}^c$ (nm)	$\Phi_f{}^d$	$\tau_f{}^e$ (ns)
1,4-dioxane	1.37	476	517	0.08	0.36 (0.85)	0.048 (0.15)	0.35	482	518	0.081	0.35
toluene	0.59	483	510	0.06	0.20 (0.75)	0.38 (0.25)	0.27	486	508	0.035(0.044)	< 0.4[g]
ethyl acetate	0.45	483	526	0.013	0.12 (0.18)	0.018 (0.82)	0.078	488	523	0.029	---
cyclohexanol	41.1	497	530	0.11	0.51 (0.65)	0.27 (0.35)	0.46	---	---	---	---
DMSO	2.24	505	543	0.004	0.018	---	0.018	504	540	0.008(0.005)	0.026
1-butanol	2.95	501	530	0.02	0.056	---	0.056	497	528	0.023(0.023)	---
DMF	0.92	501	535	0.003	---	---	---	500	533	0.004	---
2-propanol	2.40	501	528	0.010	0.040	---	0.040	495	524	0.018(0.015)	---
acetonitrile	0.35	493	539	~0.001	< 0.01	---	< 0.01	495	530	0.002(0.001)	---
methanol	0.60	496	531	~0.001	< 0.01	---	< 0.01	496	531	0.003(0.002)	---

[a] viscosity. [b] UV/Vis absorption maximum. [c] Emission maximum. [d] Fluorescence quantum yield. Literature values for **2** in parentheses. For toluene, the initially published value from ref. [10] was corrected in ref. [11]. Since the values in the other solvents published in ref. [12] were measured relative to the incorrect value of 0.10 in toluene we multiplied the published values by 0.44. [e] Fluorescence decay times and amplitudes. [f] Average fluorescence decay time (eq. S2). [g] From ref. [12].

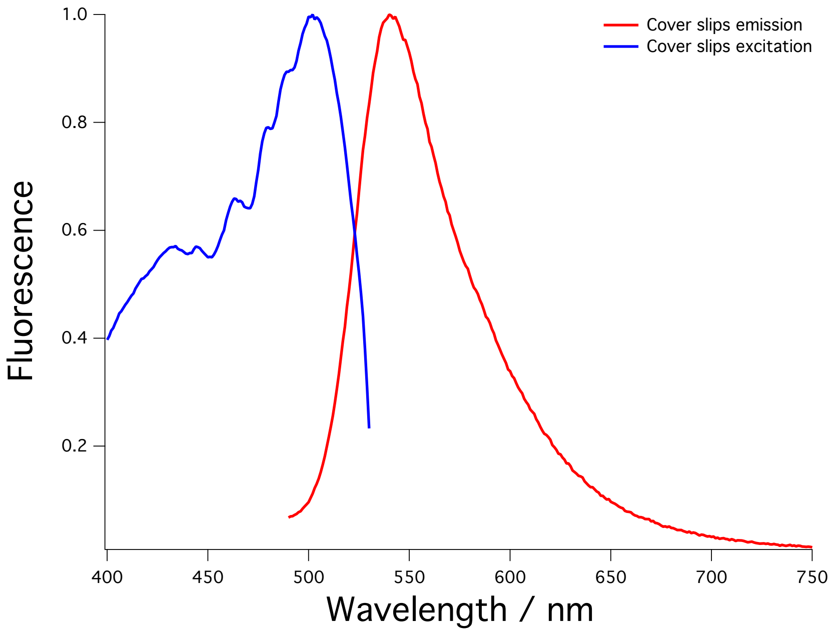

Figure S2. Excitation (blue, λ_{mon} = 550 nm) and emission (red, λ_{exc} = 450 nm) spectra of glass cover slips functionalized with **1**.

3. NMR Spectra

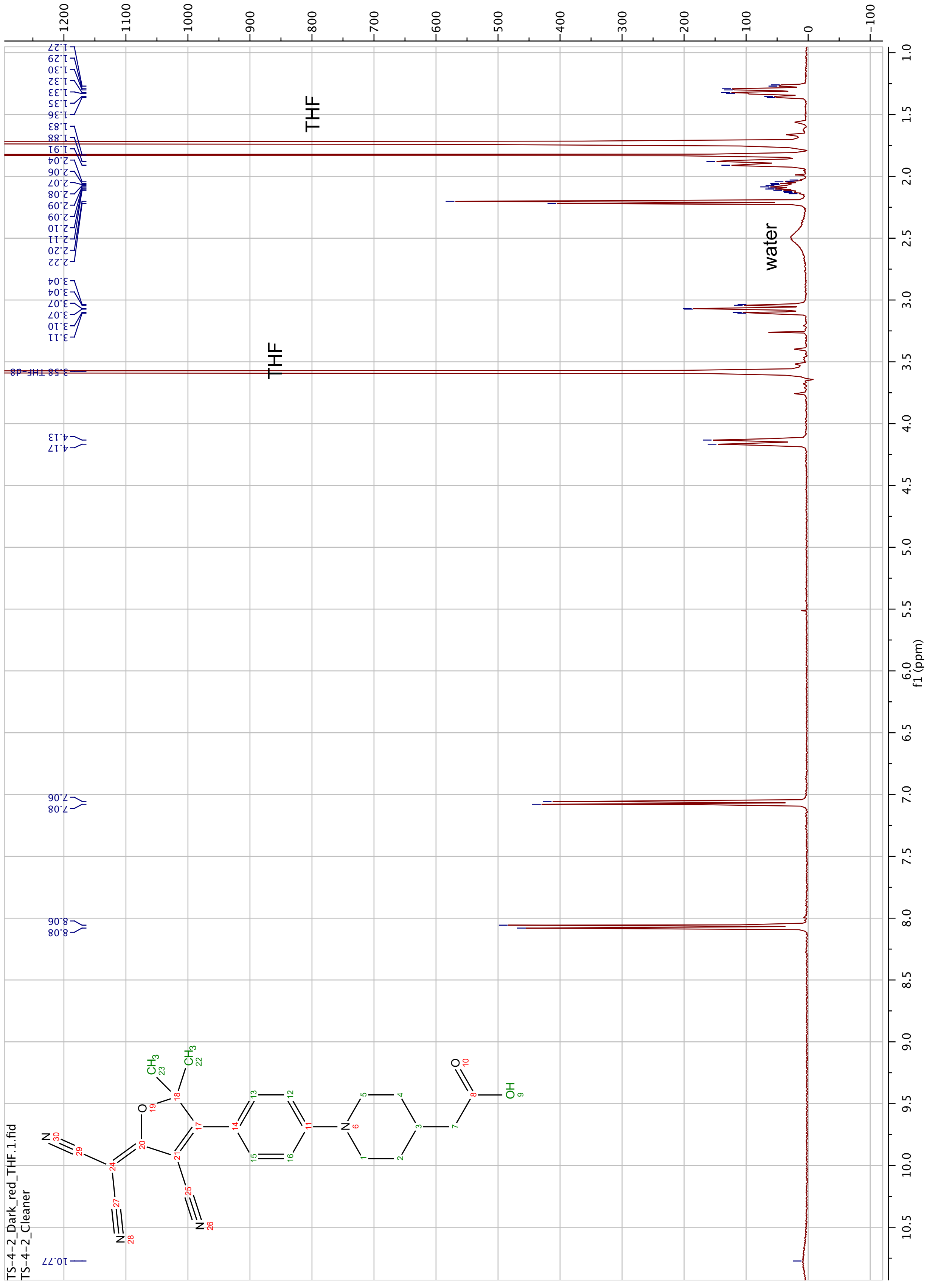

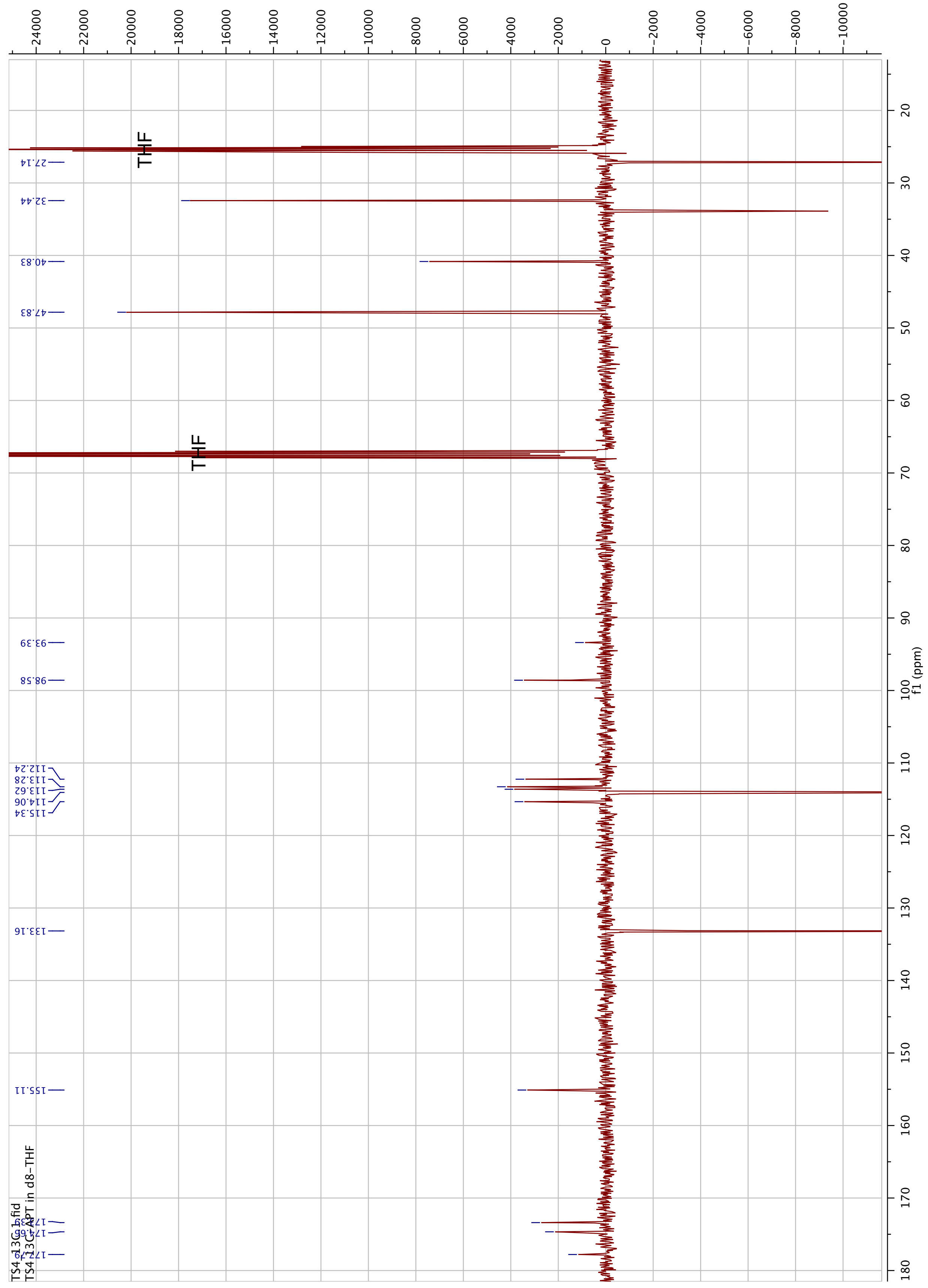
TS4 13C 1.fid
TS4 13C APT in d8-THF
THF
THF
27.14
32.44
40.83
47.83
93.39
98.58
112.24
113.28
113.62
114.06
115.34
133.16
155.11
f1 (ppm)

4. References

[1] C. Würth, M. Grabolle, J. Pauli, M. Spieles, U. Resch-Genger, *Nat. Protoc.* **2013**, *8*, 1535–1550.

[2] D. C. Jagesar, Intercomponent Interactions and Mobility in Hydrogen-Bonded Rotaxanes, PhD. Thesis, University of Amsterdam, **2010**.

[3] U. Gubler, M. He, D. Wright, Y. Roh, R. Twieg, W. E. Moerner, *Adv. Mater.* **2002**, *14*, 313–317.

[4] K. A. Willets, S. Y. Nishimura, P. J. Schuck, R. J. Twieg, W. E. Moerner, *Acc. Chem. Res.* **2005**, *38*, 549–556.

[5] S. J. Lord, N. R. Conley, H. L. D. Lee, S. Y. Nishimura, A. K. Pomerantz, K. A. Willets, Z. K. Lu, H. Wang, N. Liu, R. Samuel, R. Weber, A. Semyonov, M. He, R. J. Twieg, W. E. Moerner, *ChemPhysChem* **2009**, *10*, 55–65.

[6] L. Basabe-Desmonts, J. Beld, R. S. Zimmerman, J. Hernando, P. Mela, M. F. Garcia-Parajo, N. F. van Hulst, A. van den Berg, D. N. Reinhoudt, M. Crego-Calama, *J. Am. Chem. Soc.* **2004**, *126*, 7293–7299.

[7] A. van Driel, I. Nikolaev, P. Vergeer, P. Lodahl, D. Vanmaekelbergh, W. Vos, *Phys. Rev. B: Condens. Matter* **2007**, *75*, 035329.

[8] B. Valeur, M. N. Berberan-Santos, *Molecular Fluorescence: Principles and Applications, 2nd Ed.*, Wiley VCH, **2012**.

[9] J. R. Lakowicz, *Principles of Fluorescence Spectroscopy, 3rd Ed.*, Springer, New York, **2006**.

[10] K. A. Willets, O. Ostroverkhova, M. He, R. J. Twieg, W. E. Moerner, *J. Am. Chem. Soc.* **2003**, *125*, 1174–1175.

[11] Z. Lu, N. Liu, S. J. Lord, S. D. Bunge, W. E. Moerner, R. J. Twieg, *Chem. Mater.* **2009**, *21*, 797–810.

[12] K. A. Willets, P. R. Callis, W. E. Moerner, *J. Phys. Chem. B* **2004**, *108*, 10465–10473.
